# Neuronal and glial dysfunction, white matter hyperintensities and cognition in ageing and Alzheimer’s disease

**DOI:** 10.1093/braincomms/fcaf068

**Published:** 2025-02-14

**Authors:** Ann J Lee, Erica Howard, Nicole Saltiel, Jasmeet P Hayes, Scott M Hayes

**Affiliations:** Department of Psychology, The Ohio State University, Columbus, OH 43210, USA; Department of Psychology, The Ohio State University, Columbus, OH 43210, USA; Department of Psychology, The Ohio State University, Columbus, OH 43210, USA; Department of Psychology, The Ohio State University, Columbus, OH 43210, USA; Chronic Brain Injury Initiative, The Ohio State University, Columbus, OH 43210, USA; Department of Psychology, The Ohio State University, Columbus, OH 43210, USA; Chronic Brain Injury Initiative, The Ohio State University, Columbus, OH 43210, USA

**Keywords:** Alzheimer’s disease, ageing, biomarkers, episodic memory

## Abstract

This cross-sectional study examined associations between multiple fluid biomarkers of neuronal and glial dysfunction (plasma neurofilament light chain, CSF growth-associated protein 43 and CSF soluble triggering receptor expressed on myeloid cells 2), total white matter hyperintensity volume and episodic memory and executive function performance in the context of Alzheimer’s disease biomarker status. A total of 563 participants (mean age = 71.9 years, standard deviation = 7.2) from the Alzheimer’s Disease Neuroimaging Initiative were classified by the amyloid-β/tau/neurodegeneration framework into no Alzheimer’s disease pathology (*n* = 176), suspected non-Alzheimer’s disease pathophysiology (*n* = 87) or Alzheimer’s disease continuum (*n* = 300) groups. Participants completed baseline neuropsychological assessment, plasma/CSF biomarker collection and MRI. Analyses explored the relative contributions of biomarkers to episodic memory and executive function performance and whether relationships varied by amyloid-β/tau/neurodegeneration group status. Across all participants, neurofilament light chain ($\hat{\beta }$**=** −0.14, *P* < 0.001) and growth-associated protein 43 ($\hat{\beta }$**=** −0.13, *P* < 0.001) were the strongest biomarkers associated with episodic memory performance, such that greater levels were associated with worse episodic memory. There was a group by growth-associated protein 43 interaction with episodic memory: greater growth-associated protein 43 was associated with lower episodic memory performance in participants classified as Alzheimer’s disease continuum relative to the no Alzheimer’s disease pathology group ($\hat{\beta }$**=** −0.26, *P* < 0.001). No robust associations between biomarkers and executive function performance or between soluble triggering receptor expressed on myeloid cells 2, white matter hyperintensity volume and cognition were observed. Biomarkers of neuro-axonal injury and synaptic dysfunction may independently contribute to episodic memory performance across participants with differing amyloid-β/tau/neurodegeneration profiles. Growth-associated protein 43 may predict worse episodic memory performance in participants with greater Alzheimer’s disease pathology. These biomarkers of neuronal dysfunction may serve as domain-specific cognitive correlates in the context of Alzheimer’s disease biomarker status.

## Teaser

Lee *et al*. reported that biomarkers of brain dysfunction (neurofilament light chain, growth-associated protein 43) were linked to memory performance. Higher levels of growth-associated protein 43 were associated with worse memory in adults with Alzheimer’s disease. These biomarkers may be important correlates of different kinds of cognitive function (memory and attention).

## Graphical Abstract

**graphical abstract:**
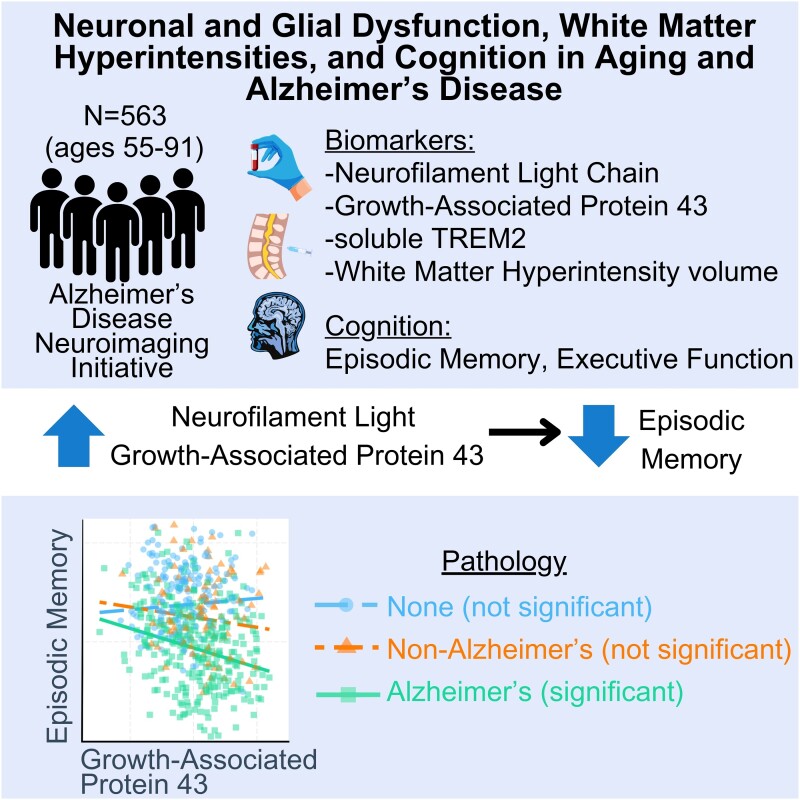

## Introduction

Alzheimer’s disease is the most common neurodegenerative disorder, characterized by widespread grey matter atrophy, distinct pathological hallmarks including amyloid-β deposition and hyperphosphorylated tau accumulation and cognitive and functional decline.^1,2^ In 2018, the National Institute on Aging and Alzheimer’s Association Research Framework proposed the amyloid-β/tau/neurodegeneration [AT(N)] classification system for Alzheimer’s disease pathogenesis, grouping core Alzheimer’s disease biomarkers by amyloid-β pathology (A), phosphorylated tau (p-tau) pathology (T) and neurodegeneration (N).^3^ However, Alzheimer’s disease pathology often does not occur in isolation,^4^ and there is extensive evidence for other processes, such as neuronal injury, synaptic loss, inflammation and cerebrovascular injury, that are associated with cognitive impairment in ageing and Alzheimer’s disease.^5-8^

Neuronal injury and dysfunction are important processes in the pathogenesis of Alzheimer’s disease and are associated with cognitive decline.^3^ One fluid biomarker of neuronal dysfunction is neurofilament light (NfL) chain. NfL is a support protein within large, myelinated axons in cerebral white matter and is considered a biomarker of neuro-axonal injury, a pathological change that is most proximal to Alzheimer’s disease symptom onset and disease progression.^3,9-11^ Higher levels of CSF and plasma NfL have been associated with greater severity of global cognitive impairment.^11-15^ Other studies have related CSF or plasma NfL to specific cognitive domains, including executive functions,^15,16^ attentional control^17^ and episodic memory performance^16,18^ among cognitively normal, mild cognitive impairment (MCI) and Alzheimer’s disease samples. Another study by Aschenbrenner *et al*.^17^ reported that CSF NfL was associated with decline in attention and global cognition but not episodic memory. Given these inconsistent findings between studies, the relationships between NfL and performance in specific cognitive domains remain unclear.

Synaptic loss and dysfunction is another early feature of Alzheimer’s disease pathology closely related to cognitive impairment.^3,19-22^ One marker of pre-synaptic degeneration and dysfunction is growth-associated protein 43 (GAP-43), an intra-cellular membrane-bound protein encoded by the GAP-43 gene. GAP-43 is essential for pre-synaptic terminal and axonal growth as well as learning and memory functions, all of which play a pivotal role in neuronal development, synaptogenesis and hippocampal function.^6,23,24^ Among patients diagnosed with MCI or Alzheimer’s disease, CSF GAP-43 levels have been shown to negatively correlate with global cognition.^6,20,25,26^ Studies using transgenic mice have also associated the overexpression of the GAP-43 protein with memory dysfunction,^27^ but limited literature has examined the associations between CSF GAP-43 and specific cognitive domains in humans.

Immune system alterations are also important in the pathogenesis of Alzheimer’s disease and relate to cognitive performance.^5,28,29^ CSF soluble triggering receptor expressed on myeloid cells 2 (sTREM2) is the extracellular domain of the TREM2 protein released into the interstitial fluid of the brain and a biomarker of microglial activation.^28^ CSF sTREM2 levels increase in pre-clinical stages of Alzheimer’s disease, indicating an early microglial response to tau pathology or neuronal injury, but decrease in later Alzheimer’s disease stages.^5,30,31^ Previous work has related higher baseline levels and annual rates of increase of CSF sTREM2 to a slower decline in global cognition, episodic memory and hippocampal atrophy in patients with Alzheimer’s disease.^5,28,29^ Therefore, greater sTREM2 concentration may reflect a healthy and protective microglial response mechanism acting against cognitive decline in early symptomatic stages of Alzheimer’s disease.

In addition to fluid biomarkers related to cognitive impairment, MRI allows for the quantification of neurovascular contributions to cognition. White matter hyperintensities (WMHs) indicate ischaemic, microstructural white matter damage and axonal degradation.^32,33^ Although the pathogenesis of WMH has been traditionally attributed to small vessel disease,^33,34^ WMH in patients with Alzheimer’s disease may be a consequence of Alzheimer’s disease and tau pathology.^33,35^ Regardless of the mechanisms of the manifestation of WMH, greater WMH load confers risk for the onset and severity of Alzheimer’s disease–associated cognitive decline,^7,36,37^ and this relationship is postulated to be dose dependent.^34^ Among older adults with MCI and Alzheimer’s disease, total WMH volume has been consistently associated with declines in executive functions and processing speed^7,36-39^ as well as episodic memory performance.^7,36,37^ Thus, WMH volume may complement fluid biomarkers to explain additional variations in cognitive performance in ageing and Alzheimer’s disease samples.

Accumulating evidence linking these biomarkers (NfL, GAP-43, sTREM2 and WMH) with cognitive decline has occurred across independent studies, with few studies simultaneously assessing these biomarkers within the same study. It is important to address this gap in the literature to better understand which biomarkers are most strongly associated with cognition and whether these associations with cognition are domain specific. Therefore, the first aim of the study was to examine the associations between multiple fluid biomarkers of neuronal and glial dysfunction (plasma NfL, CSF GAP-43 and CSF sTREM2) and WMH volume with episodic memory and executive function performance, two cognitive domains that are predominantly affected in early Alzheimer’s disease.^40-42^ To assess which biomarkers had the strongest associations with cognition across all participants, we implemented a novel relative importance analysis, which controls for order of variable entry in the regression analysis, as well as hierarchical linear regression models. Given that these biomarkers reflect different pathological mechanisms, differ in assessment methodology (plasma, CSF or MRI) and may be associated with distinct cognitive domains, this approach allowed for the assessment of differential associations among biomarkers and domain-specific cognitive performance. Based on prior work, we hypothesized that biomarkers of neuronal (plasma NfL and CSF GAP-43) and glial dysfunction (CSF sTREM2) would be associated with episodic memory performance,^16,18,27,28^ whereas biomarkers associated with axonal function (plasma NfL and WMH volume) would be associated with executive function performance.^15,16,39^

The second aim of the study was to assess the independent contributions of relatively important biomarkers (plasma NfL, CSF GAP-43, CSF sTREM2 or WMH volume) to cognitive function and whether associations were dependent on AT(N) group status. We predicted that AT(N) group status would moderate these relationships, such that older adults with pathological levels of hallmark Alzheimer’s disease biomarkers would have a stronger negative relationship between biomarker measures and cognitive function compared with older adults without pathological Alzheimer’s disease.

## Materials and methods

Data for this study were obtained from the Alzheimer’s Disease Neuroimaging Initiative (ADNI) database (adni.loni.usc.edu). The ADNI was launched in 2003 as a public–private partnership, led by Principal Investigator Michael W. Weiner, MD. The primary goal of ADNI has been to test whether serial MRI, PET, other biological markers and clinical and neuropsychological assessment can be combined to measure the progression of MCI and early Alzheimer’s disease. Extensions of the ADNI project, ADNI-Grand Opportunity (ADNI-GO) and ADNI-2 were developed with aims to assess biomarkers at earlier stages of Alzheimer’s disease. Study procedures were approved by the Institutional Review Boards of all participating institutions, and informed written consent was obtained from all participants at each site.

### Participants

Participants were selected from ADNI-GO and ADNI-2 based on available baseline plasma NfL, CSF GAP-43, CSF sTREM2, WMH volume and composite episodic memory and executive function data. The final sample included in the analyses consisted of 563 participants aged 55–91 years, classified by ADNI as cognitively normal (*n* = 119), significant memory concern (*n* = 43), early MCI (*n* = 194), late MCI (*n* = 122) or Alzheimer’s disease dementia (*n* = 85). The ADNI inclusion criteria for these clinically classified groups are presented in Supplementary Table 1. Exclusion criteria included, but were not limited to, any significant neurologic disease other than Alzheimer’s disease, known structural brain abnormalities and significant systemic illness or unstable medical conditions. Additionally, ADNI required that all participants have a Geriatric Depression Scale score <6 and a Hachinski Ischaemic Score ≤4. The full inclusion/exclusion and diagnostic criteria can be found in the ADNI procedures manual (https://adni.loni.usc.edu/wp-content/uploads/2008/07/adni2-procedures-manual.pdf).

### Plasma and CSF biomarkers

Plasma NfL was analysed at the Clinical Neurochemistry Laboratory at the University of Gothenburg, Sweden. Analysis was conducted using the single molecule array (Simoa) technique with an assay that utilized a combination of monoclonal antibodies and purified bovine NfL as a calibrator. CSF GAP-43 was measured by an in-house enzyme-linked immunosorbent assay (ELISA) method at the Clinical Neurochemistry Laboratory at the University of Gothenburg, Sweden. The ELISA assay range of CSF GAP-43 was 312–20 000 pg/mL. CSF sTREM2 was analysed using the ELISA assay by the Haass group, and CSF levels were corrected by plate-specific correction factors. All biomarker analyses were validated by ADNI, and further details are described elsewhere.^15,43,44^ Two missing CSF sTREM2 values and one missing plasma NfL value were imputed using each biomarker’s sample median value.

CSF amyloid-β (Aβ_42_) and p-tau (181P) were analysed at the UPenn/ADNI Biomarker Laboratory using Roche Elecsys. Quality control to meet the acceptance criteria for precision and accuracy was performed. The analyte measuring ranges with lower and upper technical limits were 200–1700pg/mL for Elecsys β-Amyloid CSF immunoassay and 8–120 pg/mL for Elecsys Phospho-Tau CSF immunoassay. The Elecsys β-Amyloid CSF immunoassay in use is not a commercially available *in vitro* diagnostic assay. It is an assay that is currently under development and for investigational use only. The performance of the assay beyond the upper technical limit has not been formally established. Therefore, use of values above the upper technical limit, which are provided based on an extrapolation of the calibration curve, is restricted to exploratory research purposes and is excluded for clinical decision-making or for the derivation of medical decision points. Details on the immunoassay methodologies can be found on the ADNI website (https://adni.loni.usc.edu/).

### White matter hyperintensities

Participants completed whole-brain MRI on 3-Tesla scanners. All ADNI sites followed a standardized protocol to obtain MRI data^45^ and underwent quality control at the Mayo Clinic. WMH measurement was conducted at the University of California at Davis. In brief, the measurement approach is a Bayesian approach to segmentation of high-resolution 3D T1 and fluid attenuation inversion recovery (FLAIR) sequences. Non-brain structures were excluded from 3D T1 images prior to measurement using an automated atlas-based method, and FLAIR images were affine transformed to the 3D T1 image. FLAIR inhomogeneity corrections were performed after co-registration of the FLAIR to the 3D T1 image. For further details, see the associated methods paper (https://files.alz.washington.edu/documentation/adni-proto.pdf). Total WMH volume (cm^3^) was obtained for each participant and adjusted for intracranial volume to correct for individual differences in head size.

### Episodic memory and executive function assessment

All participants completed a neuropsychological assessment at the baseline visit. We used neuropsychological composite scores of episodic memory and executive function that have been validated in individuals in the ADNI cohort.^46,47^ Normalized composite measures were derived from an iterative process that applied item response theory and confirmatory factor analysis using baseline data from the ADNI neuropsychological battery. Advantages of the use of composite scores include increased measurement precision, limited number of statistical tests needed to analyse each constituent part separately and a minimized effect of potential outlying performance on a single test.^46-48^

The episodic memory composite for ADNI-GO and ADNI-2 was comprised of the Rey Auditory Verbal Learning Test, the cognitive sub-scale of the Alzheimer’s Disease Assessment Scale, Logical Memory I (Immediate) and II (Delay) and three word recall items from the Mini-Mental State Examination (MMSE). The executive function composite for ADNI-GO and ADNI-2 included Category Fluency (animals), Trail Making Test parts A and B and five Clock drawing items (circle, symbol, numbers, hands and time). For further details on the composite scores, see the studies by Crane *et al*.^46^ and Gibbons *et al*.^47^ or the associated methods paper (ADNI_Methods_UWNPSYCHSUM.pdf).

### *APOE*-ε4 data

*APOE*-ε4 genotyping was performed at the time of participant enrolment. Genotyping was performed using the Illumina Human OmniExpress BeadChip. Participants were categorized as non-carriers (no *APOE*-ε4 allele) or carriers (one or two *APOE*-ε4 alleles). Full details on *APOE*-ε4 genotyping can be found on the ADNI website (https://adni.loni.usc.edu/).

### 18F-fluorodeoxyglucose-PET data

All participants enrolled in ADNI-GO and ADNI-2 completed 18F-fluorodeoxyglucose-positron emission tomography [(18F)FDG-PET] scans at baseline. Data were analysed at the UC Berkeley and Lawrence Berkeley National Laboratory and validated by ADNI.^49^ The region of interest (ROI)–based data analysis was conducted by identifying important hypometabolic regions indicative of pathological metabolic change in MCI and Alzheimer’s disease. Five ROIs (left angular gyrus, right angular gyrus, bilateral posterior cingular, left inferior temporal gyrus and right inferior temporal gyrus) were identified based on coordinates frequently cited in FDG studies comparing healthy, MCI and Alzheimer’s disease subjects. A composite ROI was generated by averaging across the five individual ROIs for each subject. Each ROI mean was intensity normalized by dividing the value by the pons/vermis reference region mean. Development of the ROIs is described elsewhere^49-51^ and in the associated methods paper (ADNI_UC_Berkeley_FDG_Methods_20121022.pdf).

### National Institute on Aging and Alzheimer’s Association amyloid-β/tau/neurodegeneration classification

The AT(N) research framework categorizes common neuropathological changes in Alzheimer’s disease into three groups: amyloid-β pathology (A), phosphorylated tau pathology (T) and neurodegeneration [including CSF total tau, brain atrophy measured using MRI and PET; (N)]. In the present study, the AT(N) framework was used to dichotomize participants by pathological (+) or non-pathological (−) biomarker levels using CSF Aβ_42_ (A), CSF p-tau (P181) (T) and FDG-PET (N) values. Due to the high multi-collinearity observed between CSF p-tau (‘T’ biomarker) and total tau (‘N’ biomarker; *r* = 0.98), FDG-PET was used as an alternative measure of neurodegeneration (‘N’ biomarker). Lower FDG-PET indicates neuronal hypometabolism due to neurodegeneration and was labelled as an ‘N’ biomarker in the AT(N) framework.^3^

AT(N) biomarker profiles were determined by applying published cut-off values to each biomarker.^52^ CSF Aβ_42_ abnormality (A+) was defined as <977 pg/mL. CSF p-tau abnormality (T+) was defined as >27 pg/mL. Neurodegeneration (N+) was defined using FDG-PET composite ROI <1.21 pg/mL. Eight biomarker profiles were created for each subgroup as previously described.^3^ Due to the small sample size of several profiles [A−T−(N+), *n* = 39; A−T+(N−), *n* = 23; A−T+(N+), *n* = 25] and to ensure an adequate sample size for analyses, biomarker profiles were combined to create three AT(N) groups: (i) no Alzheimer’s disease pathology [A−T−(N−), *n* = 176], (ii) suspected non-Alzheimer’s disease pathophysiology [SNAP; A−T±(N+) or A−T+(N±), *n* = 87] and (iii) Alzheimer’s disease continuum [A+T±(N±), *n* = 300]. A schematic of the classification of AT(N) groups is shown in Supplementary Table 2. See Supplementary Table 3 for details on the reclassification of ADNI clinical groups according to the AT(N) framework.

### Statistical analysis

All statistical analyses were conducted using R Studio (version 4.1.1). Demographic, biomarker, neuroimaging and cognitive data were downloaded from the ADNI database (http://adni.loni.usc.edu/). Given the association between WMH and vascular disease risk,^34^ information related to cardiometabolic health (history of smoking, hypertension, diabetes and hyperlipidaemia) was obtained using participant medical history documentation.

Prior to analysis, normality assumptions for all variables were visually inspected using Q–Q plots and evaluated using Shapiro–Wilk’s test. All biomarkers of interest (NfL, GAP-43, sTREM2 and WMH) were logarithmically transformed to approximate a normal distribution prior to analyses. Potential multi-collinearity issues were assessed using variance inflation factors (VIFs) to verify that all variables were within an acceptable range. VIF values between p-tau and CSF GAP-43 exceeded the conservative threshold of 2.5, and Pearson’s correlation coefficients confirmed the presence of moderate multi-collinearity between p-tau and GAP-43 (*r* = 0.70). Multi-collinearity in hierarchical linear regressions leads to unstable regression coefficients and problems of identification and interpretation of the separate effects of the independent variables on the response variable of interest.^53^ Given well-established relationships between p-tau and cognition in Alzheimer’s disease^3,54^ and between p-tau and CSF GAP-43,^26^ we chose to examine the association between GAP-43 and cognition in participants stratified by p-tau [‘T’ biomarker in the AT(N) classification system]. Biomarker and cognitive data outliers that were ±3 SD from the mean were removed from the final sample.

Descriptive statistics for baseline sample characteristics were assessed using chi-square (χ^2^) and one-way ANOVA tests. *Post hoc* inter-group differences were assessed using Tukey’s Honestly Significant Difference (HSD) tests. All variables were standardized prior to analyses. The alpha level for all tests was set as *P* < 0.05.

#### Relative importance of biomarker variables to cognition

General linear models were employed to assess the contributions of biomarkers of neuronal and glial dysfunction and WMH volume to episodic memory and executive function performance across all participants. Relative importance metrics, which refer to the quantification of an individual regressor’s contribution (*R*^2^) to a regression model, were computed for all variables using the recommended Lindeman, Merenda and Gold (‘lmg’) method from the *relaimpo* package in R.^55^ The ‘lmg’ metric decomposes the model-explained variance to quantify the average contribution of each predictor variable by averaging over different orders of entry into the model to control for the dependence on orderings (the order in which variables are included in the model).^55^ Although the *relaimpo* analysis can identify the relative importance (average contribution) of each predictor variable, it does not implement significance testing.

Two relatively importance analyses examined the contributions of each biomarker (NfL, GAP-43, sTREM2 and WMH) to episodic memory and executive function performance. In both models, biomarkers were entered as individual regressors, controlling for demographic variables (age, sex and years of education), *APOE-*ε4 status, vascular covariates and AT(N) group status, with episodic memory or executive function composite scores as the dependent variable. Relatively important variables of cognition were defined as lmg > 1.00. This analysis approach examined which biomarkers accounted for the most variance in cognition and informed subsequent statistical testing.

#### Associations between relatively important biomarkers and cognition

To examine whether relatively important biomarkers of interest were significantly associated with cognitive performance (which *relaimpo* does not do) across all participants, two hierarchical linear regression models were employed using identical control variables, relatively important biomarkers of cognition (defined as lmg > 1.00) and composite episodic memory or executive function scores as the dependent variable. Hierarchical linear regression models included age, sex, years of education, *APOE*-ɛ4 status, vascular covariates and AT(N) group as control variables (Step 1). Next, relatively important biomarkers of cognition (NfL, GAP-43, sTREM2 or WMH) were added as biomarker variables (Step 2). Given that we did not have *a priori* hypotheses regarding the ordered entry of biomarker variables into each model, relatively important biomarkers were entered simultaneously in Step 2. This approach thereby allowed interpretations of the coefficients of each biomarker in the context of other biomarker variables.

#### Relationships between relatively important biomarkers and cognition by amyloid-β/tau/neurodegeneration group

To examine the independent contributions of each relatively important biomarker to cognition and whether these associations varied by AT(N) group status, separate hierarchical linear regression models were run with one biomarker as the independent variable in each model and episodic memory or executive function as the dependent variable. In each model, control variables were entered in Step 1 and the respective relatively important biomarker (NfL, GAP-43, sTREM2 or WMH) was entered in Step 2. To test for potential interaction effects between each biomarker and AT(N) group status on cognition, the biomarker × AT(N) group interaction term was added in the final step of each model (Step 3). The AT(N) group variable was dummy coded with the no Alzheimer’s disease pathology group as the reference group. For each model, regression coefficients ($\hat{\beta }$), variance explained (*R*^2^) and change in variance explained (△*R*^2^) were calculated to examine the amount of variance in cognition explained at each step. Significant interaction effects were probed with a simple slopes analysis and pairwise differences of simple slopes using the *emmeans* package in R.^56^ Tukey’s HSD-adjusted *P*-values were implemented for *post hoc* comparisons.

## Results

### Study sample characteristics

Baseline study sample characteristics of participants are presented in Table 1. The mean age was 71.9 years (SD = 7.2), and the mean educational attainment was 16.2 years (SD = 2.6). Significant differences between AT(N) groups were found for all demographic, cognitive and biomarker variables (*P*s < 0.05; Table 1) except educational attainment. *Post hoc* Tukey HSD tests confirmed that participants in the SNAP and Alzheimer’s disease continuum groups were older than those in the no Alzheimer’s disease pathology group (Table 1). Participants in the Alzheimer’s disease continuum group had a higher prevalence of *APOE*-ε4 allele carriers, lower MMSE scores, lower episodic memory and executive function composite scores, lower CSF amyloid-β (Aβ_42_) and lower FDG-PET measures relative to both no Alzheimer’s disease pathology and SNAP groups. Both SNAP and Alzheimer’s disease continuum groups had greater CSF p-tau (181P) and CSF GAP-43 levels relative to the no Alzheimer’s disease pathology group. Greater total WMH volume and higher plasma NfL levels were observed in the Alzheimer’s disease continuum group compared with the no Alzheimer’s disease pathology group. In addition, the SNAP group had the highest CSF sTREM2 levels (Table 1).

**Table 1:** Baseline demographic and biomarker variables of participants

	Total (*N* = 563)	No Alzheimer’s disease pathology (*n* = 176)	SNAP (*n* = 87)	Alzheimer’s disease continuum (*n* = 300)	*Post hoc*
Age^a^	71.9 (±7.2)	69.3 (±6.5)	74.3 (±6.6)	72.7 (±7.4)	SNAP > no Alzheimer’s disease, ***P <* 0.001** Alzheimer’s disease > no Alzheimer’s disease, ***P* < 0.001** Alzheimer’s disease > SNAP, *P* = 0.15
Sex, *n* (% F)^b^	251 (45%)	92 (52%)	37 (43%)	122 (41%)	SNAP < no Alzheimer’s disease, *P* = 0.14 Alzheimer’s disease < no Alzheimer’s disease, ***P* = 0.01** Alzheimer’s disease < SNAP, *P* = 0.76
Education^a^	16.2 (±2.6)	16.3 (±2.7)	16.3 (±2.4)	16.2 (±2.7)	ns
*APOE*-ε4 carrier, *n* (%)^b^	262 (47%)	39 (22%)	26 (30%)	197 (66%)	SNAP > no Alzheimer’s disease, ***P* = 0.03** Alzheimer’s disease > no Alzheimer’s disease, ***P* < 0.001** Alzheimer’s disease > SNAP, ***P* < 0.001**
MMSE^a^	27.6 (±2.5)	28.8 (±1.5)	28.3 (±2.0)	26.7 (±2.8)	SNAP < no Alzheimer’s disease, *P* = 0.20 Alzheimer’s disease < no Alzheimer’s disease, ***P* < 0.001** Alzheimer’s disease < SNAP, ***P* < 0.001**
Episodic memory composite (*Z*-score)^a^	0.36 (±0.90)	0.97 (±0.64)	0.60 (±0.80)	−0.06 (±0.84)	SNAP < no Alzheimer’s disease, ***P* < 0.001** Alzheimer’s disease < no Alzheimer’s disease, ***P*****<0.001** Alzheimer’s disease < SNAP, ***P* < 0.001**
Executive function composite (*Z*-score)^a^	0.34 (±1.02)	0.88 (±0.79)	0.64 (±0.92)	−0.07 (±0.99)	SNAP < no Alzheimer’s disease, *P* = 0.09 Alzheimer’s disease < no Alzheimer’s disease, ***P* < 0.001** Alzheimer’s disease < SNAP, ***P* < 0.001**
CSF Aβ_42_ (pg/mL)^a^	1037 (±455)	1489 (±241)	1408 (±280)	663 (±179)	SNAP < no Alzheimer’s disease, ***P* = 0.01** Alzheimer’s disease < no Alzheimer’s disease, ***P* < 0.001** Alzheimer’s disease < SNAP, ***P* < 0.001**
CSF p-tau (pg/mL)^a^	26 (±14)	18 (±5)	29 (±13)	31 (±15)	SNAP > no Alzheimer’s disease, ***P* < 0.001** Alzheimer’s disease > no Alzheimer’s disease, ***P* < 0.001** Alzheimer’s disease > SNAP, *P* = 0.29
FDG-PET ROI (*Z*-score)^a^	1.22 (±0.17)	1.35 (±0.10)	1.19 (±0.10)	1.16 (±0.17)	SNAP < no Alzheimer’s disease, ***P* < 0.001** Alzheimer’s disease < no Alzheimer’s disease, ***P* < 0.001** Alzheimer’s disease < SNAP, *P* = 0.23
Plasma NFL (pg/mL)^a^	37.26 (±16.46)	30.21 (±13.34)	37.22 (±15.71)	41.41 (±16.95)	SNAP > no Alzheimer’s disease, ***P* < 0.01** Alzheimer’s disease > no Alzheimer’s disease, ***P* < 0.001** Alzheimer’s disease > SNAP, *P* = 0.07
CSF GAP-43 (pg/mL)^a^	5170 (±2590)	4434 (±1986)	5836 (±2658)	5409 (±2786)	SNAP > no Alzheimer’s disease, ***P* < 0.001** Alzheimer’s disease > no Alzheimer’s disease, ***P* < 0.001** SNAP > Alzheimer’s disease, *P* = 0.35
CSF sTREM2 (pg/mL)^a^	3790 (±1875)	3431 (±1542)	4841 (±2024)	3696 (±1906)	SNAP > no Alzheimer’s disease, ***P* < 0.001** Alzheimer’s disease > no Alzheimer’s disease, *P* = 0.28 SNAP > Alzheimer’s disease, ***P* < 0.001**
WMH volume (cm^3^)^a^	0.005 (±0.01)	0.003 (±0.01)	0.004 (±0.01)	0.005 (±0.01)	SNAP > no Alzheimer’s disease, *P* = 0.22 Alzheimer’s disease > no Alzheimer’s disease, ***P* < 0.001** Alzheimer’s disease > SNAP, *P* = 0.20

Values represent the mean (standard deviation) unless otherwise indicated. *P*-values in bold indicate statistical significance. Aβ_42_, Amyloid-β_42_; AD, Alzheimer's disease; CSF, cerebrospinal fluid; FDG-PET, ^18^fluorodeoxyglucose-positron emission tomography; GAP-43, growth-associated protein 43; MMSE, Mini-Mental State Examination; NfL, neurofilament light chain; P-tau, phosphorylated tau (181P); SNAP, suspected non-Alzheimer disease pathophysiology; ROI, regions-of-interest; sTREM2, soluble triggering receptor expressed on myeloid cells 2; WMH, white matter hyperintensities.

^a^Indicates *F* comparison.

^b^Indicates *χ*^2^ comparison.

### Relative importance of biomarkers variables to cognition

#### Episodic memory

**Figure 1:**
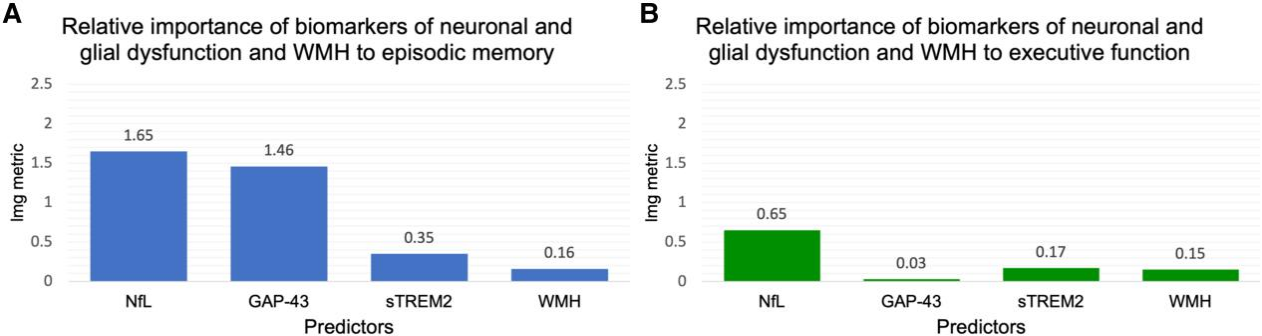
**Relative importance metrics of biomarkers to episodic memory and executive function performance derived from the relative importance analysis (*n* = 563).** (**A**) After adjusting for control variables [age, sex, years of education, *APOE*-ε4 status, vascular covariates and AT(N) group], plasma NfL and CSF GAP-43 were relatively important (lmg > 1.00) predictors of episodic memory performance. (**B**) After adjusting for control variables, biomarker variables were not relatively important predictors of executive function performance. Lindeman, Merenda and Gold (lmg) metrics represent the relative importance of each variable based on sequential *R*^2^ values and averages over order of entry into the model. AT(N), amyloid-β/tau/neurodegeneration; CSF, cerebrospinal fluid; GAP-43, growth-associated protein 43; NfL, neurofilament light chain; sTREM2, soluble triggering receptor expressed on myeloid cells 2; WMH, white matter hyperintensities.

To assess the relative importance (average contribution) among biomarkers of neuronal and glial dysfunction and WMH volume to episodic memory performance, we controlled for demographic variables, *APOE*-ε4 status, vascular covariates and AT(N) group. Control and biomarker variables explained 37.65% of the variance in episodic memory performance (range of lmg metrics = 0.05–17.53; Supplementary Fig. 1A). After adjusting for control variables, plasma NfL accounted for the most variance in episodic memory performance (lmg = 1.65) followed by CSF GAP-43 (lmg = 1.46; Fig. 1A). Lmg values reflect the average contribution of each predictor variable, independent of its position in the linear model.^55^ CSF sTREM2 and WMH volume did not have a relative importance metric above 1.00 (Fig. 1A). Thus, *post hoc* hierarchical linear regressions only included relatively important predictors (plasma NfL and CSF GAP-43; defined as lmg > 1.00) of episodic memory performance as independent regressors. For reference, the relative importance of all variables, including demographic variables, *APOE-*ε4 status, vascular covariates and biomarker variables to episodic memory performance, are displayed in Supplementary Fig. 1A.

#### Executive function

To assess the relative importance (average contribution) among biomarkers of neuronal and glial dysfunction and WMH volume to executive function performance, we controlled for demographic variables, *APOE*-ε4 status, vascular covariates and AT(N) group. Control and biomarker variables explained 28.85% of the variance in executive function performance (range of lmg metrics = 0.01–12.92; Supplementary Fig. 1B). After adjusting for control variables, plasma NfL, CSF GAP-43, CSF sTREM2 and WMH volume did not have a relative importance metric above 1.00 (Fig. 1B), suggesting that biomarker variables were not relatively important predictors of executive function performance above and beyond control variables. Given that none of the biomarkers of interest were relatively important predictors (defined as lmg > 1.00) of executive function performance, *post hoc* hierarchical linear regressions were not implemented. For reference, the relative importance of all variables, including demographic variables, *APOE-*ε4 status, vascular covariates and biomarker variables to episodic memory performance, are displayed in Supplementary Fig. 1B.

### Associations between relatively important biomarkers and cognition

*Post hoc* hierarchical linear regressions were implemented to examine whether relatively important biomarkers of episodic memory (plasma NfL and CSF GAP-43) were also significantly associated with episodic memory performance. In Step 1, control variables [demographics, *APOE*-ε4 status, vascular covariates, AT(N) group] accounted for 34% of the variance in episodic memory performance (*P* < 0.001; Table 2). In Step 2, simultaneously adding plasma NfL and CSF GAP-43 volume as independent regressors accounted for an additional 4% of the variance in episodic memory performance and improved the model (*P* < 0.001; Table 2). At Step 2 of the hierarchical regression model, sex (female, *P* < 0.001) and years of education (*P* < 0.001) were positively associated with episodic memory performance. AT(N) Alzheimer’s disease continuum group membership (*P* < 0.001), plasma NfL (*P* < 0.001) and CSF GAP-43 (*P* < 0.001) were negatively associated with episodic memory performance (Table 2).

**Table 2:** Results of hierarchical regression models examining relatively important biomarkers as predictors of episodic memory performance

Composite episodic memory (*Z*-score)						
	Step 1: Control variables			Step 2: Plasma NfL and CSF GAP-43		
Independent variable	$\hat{\beta }$	*t*	*P*	$\hat{\beta }$	*t*	*P*
Age	−0.11	−3.17	**<0.01**	−0.03	−0.67	0.50
Sex (female)	0.28	4.34	**<0.001**	0.32	4.98	**<0.001**
Education	0.17	5.29	**<0.001**	0.17	5.29	**<0.001**
*APOE*-ɛ4 status	−0.20	−2.83	**<0.01**	−0.12	−1.76	0.08
Smoking	0.07	1.10	0.27	0.09	1.40	0.16
Hypertension	−0.03	−0.14	0.89	−0.13	−0.72	0.47
Diabetes	0.42	0.80	0.43	0.51	1.00	0.32
Hyperlipidaemia	0.99	1.87	0.06	0.91	1.76	0.08
AT(N) SNAP group	−0.27	−2.64	**<0.01**	−0.19	−1.89	0.06
AT(N) Alzheimer’s disease continuum group	−0.85	−10.67	**<0.001**	−0.78	−9.71	**<0.001**
Plasma NfL (pg/mL)				−0.14	−3.79	**<0.001**
CSF GAP-43 (pg/mL)				−0.12	−3.80	**<0.001**
** ** *R* ^2^	0.34			0.38		
△*R*^2^				0.04, *F*(2,550) = 14.88, ***P* < 0.001**		
** **Model *F*	*F*(10,552) = 28.47, ***P* < 0.001**			*F*(12,550) = 27.40, ***P* < 0.001**		

Models included age, sex, education, *APOE*-ε4 status, vascular covariates and AT(N) group as control variables (Step 1), relatively important biomarkers of episodic memory performance (plasma NfL and CSF GAP-43; Step 2) and composite episodic memory as the dependent variable. After adjusting for control variables, plasma NfL and CSF GAP-43 were negatively associated with episodic memory performance and significantly improved the model. *P*-values in bold indicate statistical significance. AT(N), amyloid-β/tau/neurodegeneration; CSF, cerebrospinal fluid; FDG-PET, ^18^fluorodeoxyglucose-positron emission tomography; GAP-43, growth-associated protein 43; NfL, neurofilament light chain; P-tau, phosphorylated tau (P181).

### Relationships between plasma neurofilament light, CSF growth-associated protein 43 and cognition by amyloid-β/tau/neurodegeneration group

Two separate hierarchical linear regression models were employed to examine independent relationships between each relatively important biomarker (plasma NfL and CSF GAP-43) and episodic memory performance and whether relationships differed by AT(N) group status. In Step 1, control variables were added to each model (identical control variables are shown in Table 2, Step 1) and explained 34% of the variance in episodic memory performance. In Step 2, plasma NfL and CSF GAP-43 were entered in separate models to examine the main effect of each biomarker for predicting episodic memory performance (Table 3). Results showed that plasma NfL and CSF GAP-43 were independently associated with episodic memory performance, such that greater biomarker levels were associated with worse episodic memory performance (*P*s < 0.001; Table 3). Adding each biomarker improved their respective models, with plasma NfL and CSF GAP-43 each accounting for an additional 2% of variance in episodic memory performance (*P*s < 0.001; Table 3).

**Table 3:** Results of separate hierarchical linear regression models examining associations between relatively important biomarkers of episodic memory performance and composite episodic memory by AT(N) group

Composite episodic memory (*Z*-score)						
	Step 2: Plasma NfL (pg/mL)			Step 2: CSF GAP-43 (pg/mL)		
Independent variable	$\hat{\beta }$	*t*	*P*	$\hat{\beta }$	*t*	*P*
Age	−0.04	−0.99	0.32	−0.09	−2.75	**<0.01**
Sex (female)	0.31	4.79	**<0.001**	0.29	4.54	**<0.001**
Education	0.17	5.27	**<0.001**	0.17	5.32	**<0.001**
*APOE*-ɛ4 status	−0.17	−2.47	**0**.**01**	−0.15	−2.09	**0.04**
Smoking	0.07	1.16	0.25	0.09	1.35	0.18
Hypertension	−0.06	−0.29	0.77	−0.11	−0.57	0.57
Diabetes	0.53	1.02	0.31	0.41	0.78	0.44
Hyperlipidaemia	0.95	1.82	0.07	0.95	1.81	0.07
AT(N) group: SNAP	−0.24	−2.44	**0**.**02**	−0.21	−2.07	**0.04**
AT(N) group: Alzheimer’s disease continuum	−0.79	−9.73	**<0.001**	−0.84	−10.64	**<0.001**
Biomarker	−0.14	−3.87	**<0.001**	−0.13	−3.88	**<0.001**
** ** *R* ^2^	0.36			0.36		
△*R*^2^	0.02, *F*(1,551) = 14.94, ***P* < 0.001**			0.02, *F*(1,551) = 15.04, ***P* < 0.001**		
** **Model *F*	*F*(11,551) = 27.89, ***P* < 0.001**			*F*(11,551) = 27.91, ***P* < 0.001**		

Models included control variables (Step 1), plasma NfL and CSF GAP-43 (Step 2), the biomarker × AT(N) group interaction term (Step 3) and composite episodic memory as the dependent variable. At Step 2, Plasma NfL and CSF GAP-43 showed independent, negative associations with episodic memory performance. *P*-values in bold indicate statistical significance. AD, Alzheimer's disease; AT(N), amyloid-β/tau/neurodegeneration; CSF, cerebrospinal fluid; GAP-43, growth-associated protein 43; NfL, neurofilament light chain; SNAP, suspected non-Alzheimer disease pathophysiology.

**Figure 2:**
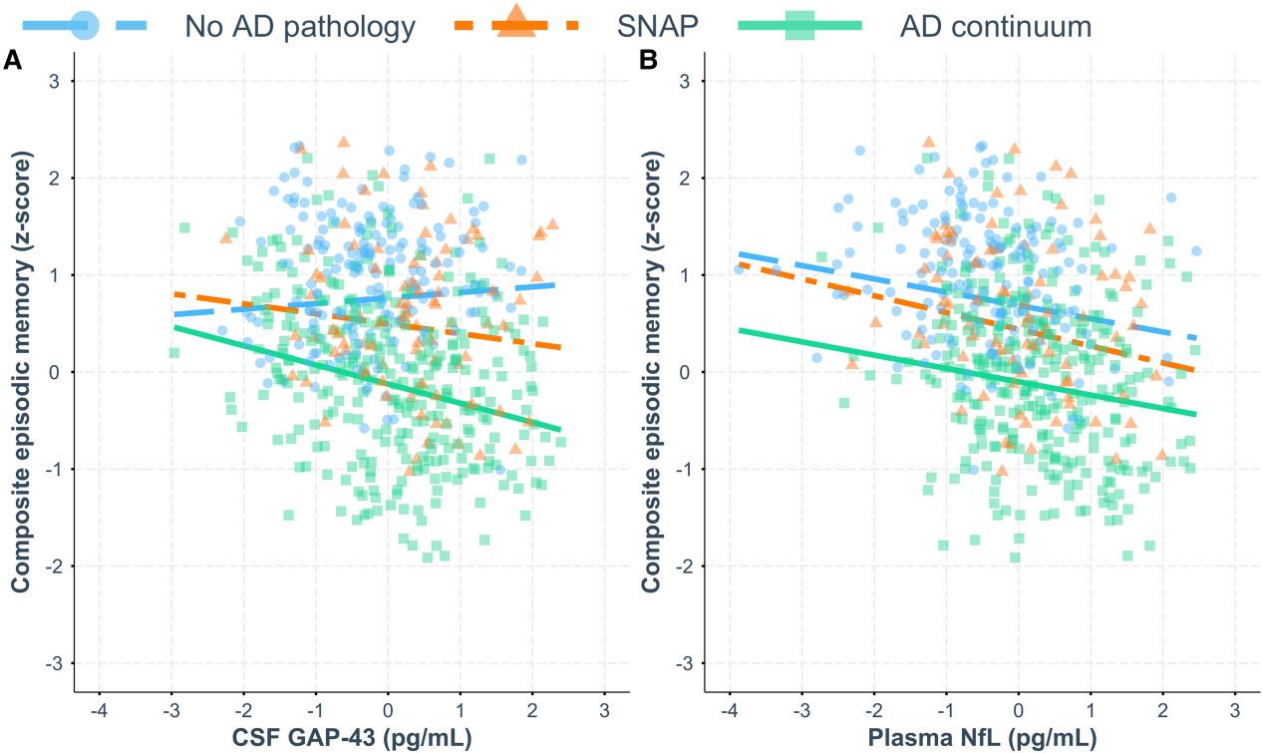
**Associations between CSF GAP-43, plasma NfL and episodic memory performance with AT(N) group as the moderating variable using hierarchical linear regression analyses (*n* = 563).** (**A**) Greater CSF GAP-43 levels were associated with worse episodic memory performance in the Alzheimer’s disease continuum group relative to the no Alzheimer’s disease pathology group [$\hat{\beta }$ = −0.26, *P* < 0.001, *F*(13,549) = 24.94]. (**B**) No significant interaction effect was observed between plasma NfL and AT(N) group on episodic memory performance. Each data point represents a participant’s CSF GAP-43 or plasma NfL level (*x*-axis) and composite episodic memory score (*y*-axis). AD, Alzheimer's disease; CSF, cerebrospinal fluid; GAP-43, growth-associated protein 43; NfL, neurofilament light chain; SNAP, suspected non-Alzheimer disease pathophysiology.

Lastly, the biomarker × AT(N) group interaction term was added to each model (Step 3). Results showed that the CSF GAP-43 × AT(N) group interaction was associated with episodic memory performance, such that greater levels of CSF GAP-43 were associated with worse episodic memory performance in the Alzheimer’s disease continuum group relative to the no Alzheimer’s disease pathology group (Table 4; Fig. 2A). Adding this CSF GAP-43 × AT(N) interaction term improved the model (*P* < 0.01; Table 4). No plasma NfL × AT(N) group interaction was observed (Table 4; Fig. 2B). *Post hoc* simple slopes analysis confirmed that CSF GAP-43 was negatively associated with episodic memory performance in the Alzheimer’s disease continuum group [95% confidence interval (CI) (−0.28, −0.12)] but not in the no Alzheimer’s disease pathology or SNAP groups (Table 5). The slope of GAP-43 on episodic memory performance in the Alzheimer’s disease continuum group was significantly different from the slope in the no Alzheimer’s disease pathology group, which was not significant (*P* < 0.01; Table 5).

**Table 4:** Results of separate hierarchical linear regression models examining associations between relatively important biomarkers of episodic memory performance and composite episodic memory by AT(N) group

Composite episodic memory (*Z*-score)						
	Step 3. Biomarker × AT(N) group interaction					
	Plasma NfL (pg/mL)			CSF GAP-43 (pg/mL)		
Independent variable	$\hat{\beta }$	*t*	*P*	$\hat{\beta }$	*t*	*P*
Age	−0.04	−0.99	0.32	−0.09	−2.76	**0**.**01**
Sex (female)	0.31	4.80	**<0.001**	0.31	4.76	**<0.001**
Education	0.17	5.24	**<0.001**	0.16	5.13	**<0.001**
*APOE*-ɛ4 status	−0.18	−2.48	**0**.**01**	−0.13	−1.81	0.07
Smoking	0.07	1.17	0.24	0.08	1.24	0.22
Hypertension	−0.05	−0.28	0.78	−0.12	−0.63	0.53
Diabetes	0.53	1.01	0.31	0.47	0.91	0.36
Hyperlipidaemia	0.95	1.82	0.07	1.00	1.94	0.05
AT(N) group: SNAP	−0.25	−2.40	**0**.**02**	−0.26	−2.56	**0**.**01**
AT(N) group: Alzheimer’s disease continuum	−0.79	−9.52	**<0.001**	−0.89	−11.16	**<0.001**
Biomarker	−0.14	−2.30	**0**.**02**	0.05	0.85	0.40
Biomarker × SNAP group	−0.04	−0.39	0.70	−0.15	−1.40	0.16
Biomarker × Alzheimer’s disease continuum group	0.00	0.00	0.99	−0.26	−3.42	**<0.001**
** ** *R* ^2^	0.36			0.37		
△*R*^2^	0.0002, *F*(2,549) = 0.09, *P* = 0.91			0.01, *F*(2,549) = 5.92, ***P* < 0.01**		
** **Model *F*	*F*(13,549) = 23.54, ***P* < 0.001**			*F*(13,549) = 24.94, ***P* < 0.001**		

Models included control variables (Step 1), plasma NfL and CSF GAP-43 (Step 2), the biomarker × AT(N) group interaction term (Step 3) and composite episodic memory as the dependent variable. At Step 3, interaction results demonstrated a significant CSF GAP-43 by Alzheimer’s disease continuum group interaction on episodic memory performance. *P*-values in bold indicate statistical significance. AD, Alzheimer's disease; AT(N), amyloid-β/tau/neurodegeneration; CSF, cerebrospinal fluid; GAP-43, growth-associated protein 43; NfL, neurofilament light chain; SNAP, suspected non-Alzheimer disease pathophysiology.

**Table 5:** *Post hoc* simple slopes analysis and pairwise differences of the simple slopes for significant interactions

Composite episodic memory (*Z*-score)		
CSF GAP-43 (pg/mL)		
	$\hat{\beta }$	*P*-adj
Simple slopes		
No Alzheimer’s disease pathology group	0.05 (−0.07, 0.18)	
SNAP group	−0.10 (−0.27, 0.07)	
Alzheimer’s disease continuum group	−0.20 **(−0.28, −0.12)**	
Pairwise differences of simple slopes		
No Alzheimer’s disease pathology—SNAP	0.15	0.34
No Alzheimer’s disease pathology—Alzheimer’s disease continuum	0.26	**<0.01**
SNAP—Alzheimer’s disease continuum	0.11	0.49

Results confirmed that CSF GAP-43 was negatively associated with episodic memory performance in the Alzheimer’s disease continuum group relative to no Alzheimer’s disease pathology group. Values in brackets indicate the 95% CI. *P-*adjusted values indicate Tukey-adjusted *P*-values. CI and *P*-values in bold indicate statistical significance. AD, Alzheimer's disease; Adj, adjusted; CSF, cerebrospinal fluid; GAP-43, growth-associated protein 43; SNAP, suspected non-Alzheimer disease pathophysiology.

## Discussion

Profiles of multiple *in vivo* fluid and neuroimaging biomarkers provide greater insight into neuropathological correlates of cognitive ageing and Alzheimer’s disease–related cognitive performance. The present study examined multiple biomarkers of neuronal and glial dysfunction (plasma NfL, CSF GAP-43 and CSF sTREM2) and WMH volume to examine which biomarkers were most strongly associated with episodic memory and executive function performance in a cohort of older adults. Additionally, we examined whether Alzheimer’s disease pathology moderated these relationships by stratifying older adults into no Alzheimer’s disease pathology, SNAP and Alzheimer’s disease continuum groups using the AT(N) framework.^3^ In the current study, across all older adults, biomarkers of neuronal dysfunction (plasma NfL and CSF GAP-43) had the highest relative importance metrics for episodic memory performance after adjusting for control variables. These results were corroborated by our hierarchical regression analyses. When plasma NfL and CSF GAP-43 were entered as simultaneous predictors of episodic memory performance in a single model, both biomarkers of neuronal dysfunction were negatively associated with episodic memory and explained additional variance in episodic memory performance. Consistent with prior studies implicating synaptic dysfunction with core Alzheimer’s disease pathologies,^57-61^ we also found that an increase in CSF GAP-43 was associated with worse episodic memory performance specifically in older adults in the Alzheimer’s disease continuum group. We did not find robust associations between biomarkers and executive function performance. Taken together, these findings suggest that biomarkers of neuronal dysfunction are relatively important correlates of domain-specific cognition across older adults and demonstrate differential associations with cognitive performance between AT(N) groups.

In the current study, plasma NfL, a biomarker of large calibre neuro-axonal injury, was a relatively important correlate of episodic memory performance across all older adults and AT(N) groups. Our findings highlight the significance of neuro-axonal injury as a mechanism contributing to deficits in episodic memory performance. Previous cross-sectional studies have likewise demonstrated a negative correlation between plasma NfL and verbal episodic memory performance among individuals with subjective cognitive decline or with pathological levels of Alzheimer’s disease biomarkers.^18,62^ Similarly, another study reported a negative relationship between plasma NfL levels and a composite episodic memory score among cognitively normal and MCI older adults.^16^ Collectively, these studies support the utility of plasma NfL as a correlate of episodic memory performance, which is consistent with our results. In contrast, plasma NfL was not identified as a relatively important predictor of executive function performance, suggesting that plasma NfL may represent a domain-specific correlate of episodic memory performance.

We did not observe evidence for a moderating role of AT(N) group status on the association between NfL and cognition, suggesting that plasma NfL may be a biomarker of episodic memory performance that operates similarly across AT(N) groups. These findings are in line with converging evidence implicating NfL as a non-specific marker of neurodegeneration,^9,15,63^ as elevated concentrations of plasma NfL have been found across various neurodegenerative diseases.^15,22,64,65^ Indeed, the association between NfL and cognitive outcomes was found to be independent of elevated amyloid-β levels among cognitively normal, MCI and Alzheimer’s disease older adults.^66^ Plasma NfL, in conjunction with Alzheimer’s disease–specific biomarkers, may provide greater insight for predicting impairment in episodic memory performance irrespective of Alzheimer’s disease pathology status.

The current study also identified CSF GAP-43, a biomarker of synaptic dysfunction, as a relatively important biomarker of episodic memory performance. We extend prior work that has related CSF GAP-43 to hippocampal volume and cognitive decline^6,26^ by demonstrating a negative association between CSF GAP-43 levels and episodic memory performance. In patients with Alzheimer’s disease, synaptic loss primarily occurs in the neocortex and hippocampus, a grey matter structure that is essential for the formation and retrieval of episodic memories, and is susceptible to early atrophy in Alzheimer’s disease.^67-69^ Consistent with this neuroanatomy, post-mortem neuropathological and PET imaging studies have shown that Alzheimer’s disease patients had reduced synaptic density and increased regional CSF GAP-43 concentration in the hippocampus compared with age-matched controls.^70-72^ Additionally, in cognitively normal, MCI and Alzheimer’s disease older adults, higher CSF GAP-43 levels were associated with smaller hippocampal volume and global cognitive decline over time.^6,26^ Importantly, our results add to this existing literature by demonstrating a unique, negative association between CSF GAP-43 and episodic memory performance that is independent of other biomarkers of neuronal degeneration (e.g. plasma NfL). Indeed, CSF GAP-43 reflects pre-synaptic dysfunction, which is one of the earliest detectable changes in neurodegenerative diseases and is postulated to occur prior to other mechanisms such as neuronal loss.^3,73^ Taken together, these findings suggested that CSF GAP-43 may be a relatively important biomarker of episodic memory decline.

Furthermore, our findings demonstrated a robust interaction between CSF GAP-43 and AT(N) group status on episodic memory performance. This interaction was driven by a negative association between GAP-43 and episodic memory performance in the Alzheimer’s disease continuum group, whereas this association was null in the no Alzheimer’s disease pathology group. It is well-established that amyloid-β and tau pathology play a critical role in synaptic dysfunction in Alzheimer’s disease,^57-60^ resulting in memory impairment.^61^ Previous studies leveraging CSF GAP-43 as a biomarker of synaptic dysfunction have positively related CSF GAP-43 with pathologic tau accumulation.^20,26,74^ CSF GAP-43 has also been postulated to be an Alzheimer’s disease–specific biomarker.^20^ Other work using the ADNI cohort associated greater CSF GAP-43 levels with a faster rate of global cognitive decline in older adults with pathological levels of amyloid-β.^25^ Consistent with prior work, our results provided support for the growing body of evidence that Alzheimer’s disease pathology differentially modifies the effect of CSF GAP-43 on performance in specific cognitive domains, such as episodic memory, specifically in older adults in the Alzheimer’s disease continuum group with presumed greater amyloidosis and tau pathology.

The current findings suggest that CSF sTREM2 and WMH volume were not relatively important predictors of episodic memory or executive function performance when compared with other biomarker variables (plasma NfL and CSF GAP-43). Prior work has shown that increases in CSF sTREM2 levels are indicative of a microglial response to tau pathology or neuronal injury, and elevations are independent of amyloidosis.^5,30,31^ Indeed, one cross-sectional study positively correlated CSF sTREM2 levels with CSF phospho-tau (181P) and total tau, suggesting that microglial-induced sTREM2 increases as tau pathology increases.^75^ Other work has suggested a protective effect of increased sTREM2, evidenced by a slower decline in global cognition, episodic memory and hippocampal atrophy in patients with Alzheimer’s disease.^5,28,29,76^ However, this protective effect is postulated to decrease as TREM2 activation and microglial function become aberrant with the progression of Alzheimer’s disease or with greater tau pathology.^5,77^ Moreover, our findings suggested that biomarkers of microglial functioning (CSF sTREM2) may be less relatively important than biomarkers of neuronal injury (plasma NfL and CSF GAP-43) as correlates of cognitive performance in groups defined by Alzheimer’s disease pathogenesis.

Consistent with prior work demonstrating the importance of Alzheimer’s disease biomarkers and the AT(N) framework to cognitive performance,^3,78,79^ unadjusted relative importance analyses identified AT(N) group as the variable accounting for the most variance in cognition in the current study, followed by plasma NfL (Supplementary Fig. 1). Importantly, plasma NfL had a greater unadjusted relative importance metric for episodic memory and executive function performance compared with demographic variables, *APOE*-ε4 status, other biomarkers of neuronal and glial dysfunction and WMH volume. Older age, female sex and *APOE*-ε4 genotype are well-established risk factors for Alzheimer’s disease.^80^ Fewer years of educational attainment have also been associated with declines in episodic memory and executive function capacities,^81,82^ which may be related to lower cognitive reserve.^83^ Moreover, our findings suggest plasma NfL as a biomarker with relatively greater contributions to cognition, compared with other biomarkers of neuronal and glial dysfunction and WMH volume, that may supplement the role of these other well-established predictors of age- and Alzheimer’s disease related cognitive impairment.

In the current study, total WMH volume was not associated with cognition despite negative associations reported in other studies using ADNI data.^37,84-86^ One reason for this discrepancy may be differences in study aims and analytic approach. Prior work using ADNI has assessed the relationship between WMH and cognition without controlling for Alzheimer’s disease biomarker status^37,85^ or other biomarkers associated with cognition (e.g. plasma NfL). Indeed, in the current study, Alzheimer’s disease biomarker status accounted for an average of 17.5% of the variance in episodic memory and nearly 13% of the variance in executive function. Given the amount of variance accounted for by Alzheimer’s disease biomarkers in the models, their inclusion in the models may have contributed to the lack of observed association between WMH and cognition in the current analysis. Inclusion of biomarkers of neuronal and glial dysfunction in our analyses may have further reduced the potential for WMH volume to account for variance in cognition. To our knowledge, only one previous study has examined the combined effect of plasma NfL and WMH volume on cognition using data from the Chicago Health and Aging Project.^87^ In that study, the authors suggested that WMH was more advantageous in predicting global cognition compared with plasma NfL.^87^ However, our findings suggested that plasma NfL may be of greater importance relative to total WMH volume for predicting performance in specific cognitive domains, such as episodic memory. Other work has suggested an increased negative effect of WMH burden on cognition in individuals with lower levels of Alzheimer’s disease pathology, whereas the impact of WMH is attenuated in the presence of greater Alzheimer’s disease–related pathologies.^84,87,88^ Thus, WMH volume may have less effect on cognition in our primarily impaired sample (*n* = 401, 71% with MCI or Alzheimer’s disease). The effect of WMH volume on cognition may also be stratified to specific AT(N) biomarker profiles, as evidenced by a previous study using the ADNI cohort.^86^

Other measures that were not assessed in the current study may share variance with the analysed biomarkers or explain additional variance in cognitive performance. For instance, structural and functional MRI studies have demonstrated associations between hippocampal atrophy,^89^ compromised microstructural and grey matter integrity^90^ and decreased regional functional connectivity^91^ with cognitive performance in non-demented, MCI and Alzheimer’s disease older adults. Other work has demonstrated that modifiable attributes of physical activity^92^ and cardiorespiratory fitness^93^ accounted for additional variance in cognition after adjusting for control variables, including sex, education and depression scores. Taken together, these studies highlight the importance of examining the associations between multiple variables such as plasma and CSF biomarkers, MRI and modifiable fitness and cognitive performance. The current findings represent an initial step in that direction, and future work may consider multivariate approaches to identify the greatest contributors to domain-specific cognitive performance.^94^

Potential clinical applications of our findings include preliminary support for plasma NfL as a potential biomarker associated with episodic memory regardless of the status of more recently established Alzheimer’s disease biomarkers, such as those within the AT(N) framework. Recent technological advancements in ultrasensitive assays suggest blood-based biomarkers as next-generation diagnostics for Alzheimer’s disease.^95,96^ Blood-based biomarkers, such as plasma NfL, provide an accessible (non-invasive, cost-effective and infrastructure independent) alternative to CSF, PET and MRI modalities.^95,96^ Plasma NfL has been correlated with CSF NfL^15,16,97^ and demonstrated similar associations with CSF NfL to MRI indices of neurodegeneration,^97^ further supporting the potential utility of plasma NfL as an indicator of episodic memory impairment and associated neurodegenerative pathology. Importantly, blood-based biomarkers may provide opportunities to increase equitable healthcare delivery and facilitate early diagnosis,^96^ which may be of particular importance given current demographic trends. In contrast, collection of CSF GAP-43 required a spinal tap, as most synaptic proteins are not yet able to be assayed in blood.^98^ Spinal taps are more invasive and less practical than other assays, such as a blood draw or brain MRI. Therefore, CSF GAP-43 is likely to have more limited clinical utility given the more invasive and less practical data collection methods. Nevertheless, our findings may be relevant for future research and clinical trials on candidate blood-based biomarkers of synaptic dysfunction and their associations with episodic memory impairment and provide mechanistic insight into Alzheimer’s disease progression.

Importantly, a sub-set of participants diagnosed as cognitively normal using ADNI’s criteria were classified in the AT(N) Alzheimer’s disease continuum group in the current study (*n* = 40, 44% of cognitively normal group). The pathophysiological process of Alzheimer’s disease occurs years prior to the manifestation of cognitive symptoms.^99^ These individuals likely had early pathological features of Alzheimer’s disease without objective memory impairment and/or subjective cognitive concerns according to the ADNI diagnostic criteria. The ADNI diagnostic criteria are limited by their reliance on singular subjective and objective cognitive measures that are prone to bias.^100^ Alternatively, the AT(N) framework provides a less biased, descriptive classification scheme to diagnose Alzheimer’s disease using pathologic biomarker data. Although the AT(N) framework does not take into account cognitive impairment (a hallmark symptom of Alzheimer’s disease that also predicts functional decline^101^), the AT(N) framework allows for the assessment of interactions among different pathologic processes (e.g. biomarkers) and cognitive symptoms^3^ as well as early identification of individuals at risk of Alzheimer’s disease.^78^

This study had several limitations. The sample was comprised of highly educated and demographically homogenous participants regarding racial, ethnic and socioeconomic status, which limits the generalizability of our findings. Inherent to using the AT(N) classification system, we dichotomized AT(N) biomarker data (CSF Aβ_42_, CSF p-tau and FDG-PET composite ROI) as +/− using cut-off values to classify participants as no Alzheimer’s disease pathology, suspected non-Alzheimer’s disease pathology or Alzheimer’s disease continuum, which may have led to loss of information. The small number of participants in several AT(N) biomarker profiles [A−T−(N)+, *n* = 39; A−T+(N)−, *n* = 23 and A−T+(N)+, *n* = 25] precluded the investigation of interaction effects across all AT(N) profiles, and we combined AT(N) profiles to gain statistical power for our analyses. The ADNI selection criterion also excludes participants with large amounts of cerebrovascular disease based on the Modified Hachinski Ischaemic scale. Hence, use of the ADNI cohort may have contributed to the lack of findings between total WMH volume and cognition in the current sample. A primary goal of the current study was to assess the relative contributions among novel fluid biomarkers and WMH volume to cognition, and therefore, regional (hippocampal volume) or network MRI (default mode network connectivity) measures were not assessed. The extent to which the current biomarkers are more or less important to cognitive performance relative to structural and functional MRI measures would be informative to assess in future studies.

Study strengths included a novel relative importance analysis to partition the relative importance of multiple plasma, CSF and neuroimaging biomarkers reflecting differing neuropathological and cerebrovascular contributions to domain-specific cognitive performance. Examining biomarkers beyond hallmark Alzheimer’s disease pathologies provides insight on concomitant neuropathological pathways in age- and Alzheimer’s disease–related cognitive decline. Our method of stratifying participants according to the AT(N) classification system provided a biomarker-based approach to assess the moderating role of AT(N) group status on these associations. Consistent with previous reports, we observed high multi-collinearity between CSF p-tau and total tau. To address this issue, we used FDG-PET as an alternative measure of neurodegeneration (‘N’ biomarker) to reduce collinearity between the ‘T’ biomarker (p-tau) and ‘N’ biomarker for generating AT(N) groups. This approach allowed for ‘A’, ‘T’ and ‘N’ biomarkers to provide independent information on Alzheimer’s disease pathology. Lastly, we evaluated biomarker associations with validated composite scores of specific cognitive domains. Composite scores have a psychometric advantage over individual tests because they do not rely on a score derived from a single test.^46-48^ Testing differential relationships with episodic memory and executive function performance, rather than using a global cognition measure or screener, is essential given that domain-specific impairment has differing consequences for the diagnosis of neurodegenerative disease as well as Alzheimer’s disease progression.^42,102^

The present study adds to the growing body of literature that implicates novel *in vivo* fluid biomarkers associated with domain-specific cognitive performance across older adult participants and between AT(N) stratified groups. Our findings revealed plasma NfL and CSF GAP-43 as relatively important biomarker correlates of episodic memory performance and suggested independent contributions of biomarkers of neuro-axonal injury and synaptic dysfunction on episodic memory. The relationship between CSF GAP-43 and episodic memory performance varied by AT(N) group status, suggesting a compounded effect of Alzheimer’s disease pathology and CSF GAP-43 on episodic memory performance in older adults in the Alzheimer’s disease continuum group. In contrast, plasma NfL may be a biomarker of episodic memory performance that operates similarly across AT(N) groups. Future studies should examine additional biomarkers of different co-pathologies and methodologies across the disease course, specifically in the context of hallmark Alzheimer’s disease pathologies and domain-specific cognitive impairment.

## Supplementary Material

fcaf068_Supplementary_Data

## Data Availability

The data supporting the findings of this study are openly available in the Alzheimer’s Disease Neuroimaging Initiative database: https://adni.loni.usc.edu/. Codes generated and used in this work are available in a data repository: https://github.com/osubbal/AD-Biomarkers-Cognition_ALee.
