# Supplementary material for: Neuronal and glial dysfunction, white matter hyperintensities and cognition in ageing and Alzheimer’s disease
Source: Brain Commun. 2025 Feb 14;7(1):fcaf068. doi: 10.1093/braincomms/fcaf068 (PMC11848269; doi:10.1093/braincomms/fcaf068)
Supplement: fcaf068_Supplementary_Data [file fcaf068_supplementary_data.docx]

**Supplementary Table 1. Alzheimer’s Disease Neuroimaging Initiative (ADNI) inclusion criteria for cognitively normal, significant memory concern, early MCI, late MCI, and Alzheimer’s disease groups.**

|  | **Cognitively normal** | **Significant memory concern** | **Early MCI** | **Late MCI** | **Alzheimer’s disease** |
| --- | --- | --- | --- | --- | --- |
| **Subjective memory complaints** | Subjects must be free of memory complaints | Significant subjective memory concern reported by subject, informant, or clinician; CCI score ≥16 (based on first 12 questions) | Subjective memory concern reported by subject, informant, or clinician | Same as Early MCI | Same as Early MCI |
| **Memory function (Logical Memory II subscale scores)** | Normal memory function documented by scoring above education adjusted cutoffs on the Logical Memory II subscale from the Wechsler Memory Scale – Revised  a. ≥ 9 for 16 or more years of education  b. ≥ 5 for 8-15 years of education  c. ≥ 3 for 0-7 years of education | Same as Cognitively Normal | Abnormal memory function documented by scoring within education adjusted cutoffs on the Logical Memory II subscale from the Wechsler Memory Scale – Revised  a. 9-11 for 16 or more years of education  b. 5-9 for 8-15 years of education  c. 3-6 for 0-7 years of education | Abnormal memory function documented by scoring below education adjusted cutoffs on the Logical Memory II subscale from the Wechsler Memory Scale – Revised  a. ≤ 8 for 16 or more years of education  b. ≤ 4 for 8-15 years of education  c. ≤ 2 for 0 -7 years of education | Same as Late MCI |
| **Mini-Mental State Examination scores** | 24-30 inclusive | Same as Cognitively Normal | Same as Cognitively Normal | Same as Cognitively Normal | 20-26 inclusive with exceptions for subjects with < 8 years of education at the discretion of the project director |
| **Clinical Dementia Rating and Memory Box scores** | Clinical Dementia Rating = 0; Memory Box score = 0 | Clinical Dementia Rating = 0 | Clinical Dementia Rating = 0.5; Memory Box score = 0.5 | Same as Early MCI | Clinical Dementia Rating = 0.5 or 1.0 |
| **General cognition and functional performance** | Absence of significant impairment in cognitive functions or activities of daily living | Non-MCI and non-demented | General cognition and functional performance sufficiently preserved | Same as Early MCI | NINCDS/ADRDA criteria for probable Alzheimer’s disease |

Abbreviations: MCI = Mild cognitive impairment, NINCDS/ADRDA = National Institute of Neurological and Communicative Diseases and Stroke/Alzheimer's Disease and Related Disorders Association.

**Supplementary Table 2. Amyloid-β/Tau/Neurodegeneration (AT[N]) classification for AT(N) groups.** Participants were classified by pathological or non-pathological levels of CSF Aβ_42_ (A), CSF p-tau (T), and FDG-PET (N).

| **AT(N) Group** | **AT(N) Classification** | **AT(N) Profile** | **Description** |
| --- | --- | --- | --- |
| No AD pathology | A-T-(N-) | A-T-(N-) | Non-pathological CSF Aβ_42_, CSF p-tau, and FDG-PET |
| Suspected non-AD pathophysiology (SNAP) | A-T±[N+] or  A-T+[N±] | A-T+(N-)  A-T-(N+)  A-T+(N+) | Non-pathological CSF Aβ_42_; Pathological CSF p-tau and/or FDG-PET |
| AD continuum | A+T±[N±] | A+T+(N+)  A+T+(N-)  A+T-(N+)  A+T-(N-) | Pathological CSF Aβ_42_; Non-pathological or pathological CSF p-tau and/or FDG-PET |

Abbreviations: Aβ_42_ = Amyloid-β_42_, AD = Alzheimer’s disease, AT(N) = Amyloid-β/Tau/Neurodegeneration, CSF = Cerebrospinal fluid, FDG-PET = ^18^F-fluorodeoxyglucose-positron emission tomography, p-tau = Phosphorylated tau (P181).

**Supplementary Table 3. Reclassification of Alzheimer’s Disease Neuroimaging Initiative (ADNI) clinical groups according to the 2018 National Institute on Aging and Alzheimer’s Association (NIA-AA) Amyloid-β/Tau/Neurodegeneration (AT[N]) classification system.**

| **ADNI clinical group** | **AT(N) group:**  **No AD pathology**  (n = 176) | **AT(N) group:**  **SNAP**  (n = 87) | **AT(N) group:**  **AD continuum**  (n = 300) |
| --- | --- | --- | --- |
| **Cognitively normal** (n = 119) | n = 54 | n = 25 | n = 40 |
| **Significant memory concern** (n = 43) | n = 25 | n = 9 | n = 9 |
| **Early MCI** (n = 194) | n = 74 | n = 33 | n = 87 |
| **Late MCI** (n =122) | n = 20 | n = 15 | n = 87 |
| **Alzheimer’s Disease** (n = 85) | n = 3 | n = 5 | n = 77 |

Abbreviations: AD = Alzheimer’s disease, AT(N) = Amyloid-β/Tau/Neurodegeneration, MCI = Mild cognitive impairment, SNAP = Suspected non-Alzheimer disease pathophysiology.

**Supplementary Fig. 1. Relative importance metrics of demographic variables, *APOE-*ε4 status, vascular covariates, and biomarker variables to episodic memory and executive function performance**. **(A)** AT(N) group was the most important predictor of episodic memory performance. **(B)** AT(N) was also the most important predictor of executive function performance. Lindeman, Merenda, and Gold (lmg) metrics represent the relative importance of each variable based on sequential R^2^s and averages over orderings. Abbreviations: AT(N) = Amyloid-β/Tau/Neurodegeneration, GAP-43 = Growth-associated protein 43, NfL = Neurofilament light chain, sTREM2 = soluble triggering receptor expressed on myeloid cells 2, WMH = White matter hyperintensities.
